# Highly Pathogenic Avian Influenza A(H5N1) Virus Infection of Indoor Domestic Cats Within Dairy Industry Worker Households — Michigan, May 2024

**DOI:** 10.15585/mmwr.mm7405a2

**Published:** 2025-02-20

**Authors:** Ramya Naraharisetti, Meghan Weinberg, Becky Stoddard, Mary Grace Stobierski, Kimberly A. Dodd, Nora Wineland, Mathew Beal, Jennifer Morse, Samantha Hatter, Dodd Sledge, Katelynn Youatt, Joseph Coyle, Jevon McFadden, Timothy M. Uyeki, Lizette O. Durand

**Affiliations:** ^1^Epidemic Intelligence Service, CDC; ^2^Bureau of Infectious Disease Prevention, Michigan Department of Health and Human Services; ^3^Mid-Michigan District Health Department, Stanton, Michigan; ^4^Michigan State University College of Veterinary Medicine, East Lansing, Michigan; ^5^Michigan Department of Agriculture and Rural Development; ^6^Division of State and Local Readiness, Office of Readiness and Response, CDC; ^7^Influenza Division, National Center for Immunization and Respiratory Diseases, CDC.

**Summary**

**What is already known about this topic?**

Outdoor cats on U.S. dairy farms have been infected with highly pathogenic avian influenza (HPAI) A(H5N1) virus; infection has not been reported in indoor cats.

**What is added by this report?**

HPAI A(H5N1) virus was detected in two indoor domestic cats with respiratory and neurologic illness that lived in homes of dairy workers but had no known direct exposure to HPAI A(H5N1)–affected farms. Both dairy workers declined testing; other household members received negative test results for influenza A.

**What are the implications for public health practice?**

Veterinarians in states with confirmed HPAI A(H5N1) in livestock should consider obtaining household occupational information, testing for influenza A viruses, and wearing personal protective equipment when evaluating companion cats with respiratory or neurologic illness. Suspected cases should be reported to public and animal health officials.

## Abstract

Highly pathogenic avian influenza (HPAI) A(H5N1) virus, clade 2.3.4.4b, genotype B3.13 infection has been documented in cats on U.S. dairy cattle farms. In May 2024, the detection of HPAI A(H5N1) virus infection in two cats that were reported to be exclusively indoor, and that had respiratory and neurologic illness in different households, prompted an investigation by the Michigan Department of Health and Human Services and Mid-Michigan District Health Department (MDHHS/MMDHD). The cats’ owners and household members were interviewed and offered testing for influenza A(H5) virus. The owner of one cat worked on a dairy farm but declined A(H5) testing; three other household members received negative A(H5) test results. The owner of the other cat lived alone and worked on multiple dairy farms transporting unpasteurized milk; this worker also reported getting splashed in the face and eyes by unpasteurized milk but declined A(H5) testing. Both workers were employed in a county known by MDHHS/MMDHD to have HPAI A(H5N1) virus, clade 2.3.4.4b, genotype B3.13–positive dairy cattle. In states with confirmed HPAI A(H5N1) in livestock, veterinary care can be aided if veterinarians obtain household members’ occupational information, especially when evaluating cats with signs of respiratory or neurologic illness. If occupational exposure to HPAI A(H5N1)-infected livestock is identified among cat owners, and their companion cats are suspected to have HPAI A(H5N1) virus infection, it is important that veterinarians contact state and federal public health and animal health officials to collaborate on joint One Health investigations and testing to protect human and animal health.

## Investigation and Results

### Identification of First Case and Public Health Notification

In May 2024, the index cat (cat 1A, one of three cats in household 1), aged 5 years, exclusively indoor, spayed female domestic shorthair, experienced decreased appetite, lack of grooming, disorientation, and lethargy, followed by progressive neurologic deterioration. On the second day of illness, the cat was evaluated at a local veterinary clinic; on the fourth day, the cat was referred to the Michigan State University (MSU) Veterinary Medical Center (VMC), a tertiary care facility with advanced diagnostic and treatment capabilities where, because of rapid disease progression, cat 1A was euthanized. Because the cat’s owner had known occupational exposure to dairy cattle, and because highly pathogenic avian influenza (HPAI) A(H5N1) virus was known to be circulating in dairy cattle on Michigan dairy farms, upon approval from the state veterinarian, cat 1A’s remains were submitted to MSU’s veterinary diagnostic laboratory (VDL) for necropsy. Brain and nasal swabs tested by reverse transcription–polymerase chain reaction (RT-PCR) were positive for influenza A(H5) virus.* Genetic sequencing results identified HPAI A(H5N1) virus, clade 2.3.4.4b, genotype B3.13. The U.S. Department of Agriculture’s National Veterinary Services Laboratories (NVSL) confirmed the sequencing results; the virus was indistinguishable from viruses circulating in Michigan dairy cattle^†^ (*1*). This identification of HPAI A(H5N1) virus infection in a domestic house cat resulted in initiation of a public health investigation by the Michigan Department of Health and Human Services and Mid-Michigan District Health Department (MDHHS/MMDHD). This activity was reviewed by CDC, deemed not research, and was conducted consistent with applicable federal law and CDC policy.^§^

### Investigation and Public Health Response — Household 1

Cat 1A lived in a household with two adults, one of whom worked on a dairy farm in a county known to have HPAI A(H5N1)–positive dairy cattle; two adolescents (adolescents 1A and 1B); and two other exclusively indoor cats (cats 1B and 1C) (Figure). Cat 1B was reported to have signs of watery, purulent eye discharge, increased respirations, and decreased appetite 4 days after onset of illness in cat 1A. MSU VMC requested that the owner obtain a swab from cat 1B because the cat was too ill to be taken into the clinic, but specimens were not submitted by the owner. Cat 1B’s illness signs were reported to have resolved 11 days after cat 1A’s illness onset. Cat 1C had no signs of illness when it arrived at MSU VMC and received negative test results for influenza A 11 days after cat 1A’s illness onset. No other indoor or outdoor pets were reported. All four household members had daily contact with all three cats in the home. Although the dairy farm where the adult household member worked was not known to be affected by HPAI A(H5N1) virus, it was located near other affected farms.^¶^ This dairy farmworker did not work directly with animals but worked on the dairy farm premises. The worker reported removing work clothes and boots outside when returning to the household; these items were then brought to a location in the home that was not accessible to the cats. The cats and household members did not consume unpasteurized milk or milk products.**

**FIGURE:**
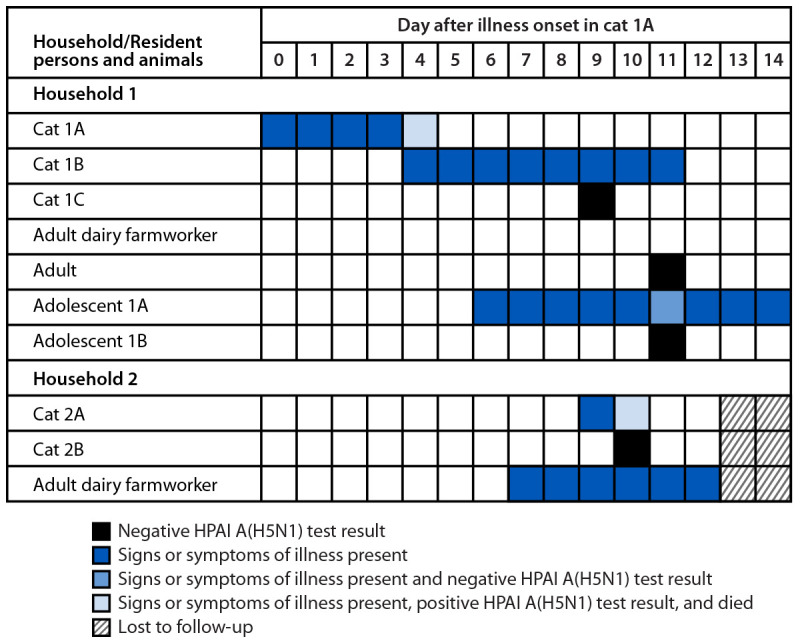
Testing, signs, and symptoms in cats*^,†^ and household members^§,¶ ^of domestic indoor cats infected with highly pathogenic avian influenza A(H5N1) virus, clade 2.3.4.4b genotype B3.13 (N = 2) — Michigan, 2024 **Abbreviation:** HPAI = highly pathogenic avian influenza. * Reported by veterinary staff members. ^†^ Sequencing of isolated influenza A virus identified virus HPAI A(H5N1) clade 2.3.4.4b genotype B3.13 in cat 1A. ^§^ Adult dairy farmworkers in households 1 and 2 received no laboratory testing. ^¶^ Adolescent 1A received a positive laboratory test result for rhinovirus/enterovirus (assay does not differentiate) on a multiplex polymerase chain reaction BioFire FilmArray Respiratory Panel (https://www.biofiredx.com/products/the-filmarray-panels/#respiratory) at the Michigan Department of Health and Human Services.

Physical examination of cat 1A by veterinary staff members was notable for signs of severe neurologic disease (obtundation, abnormality of cranial nerves, and abnormal motor function in all four limbs), anorexia, and lethargy. The cat initially had signs of ataxia, decreased appetite, swollen right jaw, abnormal gait, and hiding behavior. Some improvement and return of appetite occurred on day 3, 1 day after the cat received subcutaneous antibiotics. However, on day 4, it could not hold up its head, had an unsteady gait, and displayed all initial signs again; the cat was euthanized on day 4.

Nasopharyngeal and oropharyngeal swab specimens were collected from three household 1 members (the non farmworker adult and the two adolescents) 11 days after onset of illness in cat 1A; specimens were sent to MDHHS, where they tested negative for influenza A viruses by RT-PCR. The dairy farmworker declined influenza testing. Only one of the three persons tested (adolescent 1A, who had no comorbidities) experienced signs or symptoms of illness (cough, sore throat, headache, and myalgia), which began 6 days after illness onset in cat 1A). The household 1 members who received testing received a 10-day course of twice-daily oral oseltamivir as postexposure prophylaxis (PEP) at the time of testing; the dairy farmworker declined testing and PEP.^††^ Adolescent 1B reported a “dry croupy” cough 6 days after onset of illness in cat 1A that was attributed to severe allergies. Communication with public health representatives ended before final resolution of adolescent 1B’s symptoms was reported. Only adolescent 1A received any positive laboratory test result; this test was for rhinovirus/enterovirus on a multiplex PCR BioFire Film Array Respiratory Panel at MDHHS. Cat 1A’s owner, the dairy farmworker, had regular contact with cat 1A and adolescent 1A; the farmworker reported 1 day of vomiting and diarrhea that preceded onset of illness in cat 1A.

### Investigation and Public Health Response — Household 2

Six days after referral of cat 1A to MSU VMC, cat 2A, an exclusively indoor intact male Maine Coon cat aged 6 months from a second household (household 2), was brought by its owner directly to MSU VMC with a 1-day history of progressive neurologic deterioration, anorexia, lethargy, and facial swelling. On initial physical examination of cat 2A, it was found to be obtunded, with abnormalities of cranial nerve function, abnormal motor function, puffiness of the eyes and nose, and minimal movement; the cat died within 24 hours of onset of illness signs.

Cat 2A lived with one additional indoor cat (cat 2B) and its owner, a dairy farmworker. Nasal swabs from cat 2A tested by RT-PCR at MSU VDL, 1 day after onset of cat 2A’s illness signs and upon initial examination, were positive for influenza A viruses and were confirmed as HPAI A(H5N1) virus, clade 2.3.4.4b, genotype B3.13 at the United States Department of Agriculture’s NVSL. Cat 2B did not show any signs of illness, and nasal swabs tested negative for influenza A viruses. Cat 2A’s owner transported unpasteurized milk from various farms in a Michigan county that included farms with dairy cattle confirmed to be infected with HPAI A(H5N1) virus and lived in the same county where cat 1A’s owner lived. Cat 2A’s owner did not wear personal protective equipment (PPE) while handling raw milk; reported frequent milk splash exposures to the face, eyes, and clothing; and did not remove work clothing before entering the home when returning from work. Cat 2A’s owner reported that cat 2A would roll in the owner’s work clothes, whereas cat 2B did not exhibit this behavior. Cat 2A’s owner experienced eye irritation that began 2 days before the onset of illness signs in cat 2A but reported no other symptoms. The owner did not receive testing for influenza and declined oseltamivir and further contact with public health officials, stating fear of losing employment as a consequence of communicating with public health officials and implicating farms that provided milk.

### Screening and Testing of Veterinary Staff Members

Veterinary staff members who handled the infected cats at the local veterinary practice or MSU VMC were contacted and enrolled by public health authorities for 10 days of symptom monitoring after their last exposure to the cats. Overall, 24 veterinary staff members, including one veterinarian, five nurses, three technicians, five assistants, two caregivers, three interns, and five students, were potentially exposed to the two ill cats; 18 (75%) were contacted and monitored, but because of their limited exposure, they were not offered PEP.^§§^ PPE protocols are in place for all patients managed in MSU VMC’s isolation unit, where the cats were treated and in laboratory units where specimens were tested. For HPAI A(H5N1), recommended PPE include a Tyvek suit, boot covers, nitrile gloves, a surgical head cover, and a face mask. Veterinary staff members were reported to likely wear surgical masks for the initial encounter of cat 1A and N95 masks thereafter. Varying levels of PPE use were reported by veterinary staff members, which ranged from only using gloves to following full protocol. Laboratory staff members wear either powered air-purifying respirators or N95 respirators. Among seven persons who reported signs or symptoms after exposure to the ill cats, including four who reported nasal congestion and three who reported headache, five agreed to testing; all received negative influenza A RT-PCR test results.

## Discussion

HPAI A(H5N1) virus, clade 2.3.4.4b, has been detected in wild birds, poultry, and wildlife in the United States since 2022, and in commercial U.S. dairy cattle since 2024 (*2*–*4*). In the ongoing U.S. outbreak of HPAI A(H5N1) in dairy cattle, serious illness, including neurologic signs, and death from HPAI A(H5N1) virus infection in cats that are frequent inhabitants of farms have been attributed to consumption of unpasteurized milk from infected dairy cattle, wild birds, or raw poultry products^¶¶^ (*4*–*6*). Continued epizootic circulation of HPAI A(H5N1) virus increases the potential for emergence of mutations that might increase risk for mammalian adaption and transmission to and among humans, and this finding has been documented in the case of domestic cats (*7*). Isolated, sporadic instances of cow-to-human transmission of HPAI A(H5N1) virus, clade 2.3. 4.4b, genotype B3.13 have occurred in California, Colorado, Michigan, and Texas (*1*,*8*). Presumed cat-to-human transmission of low pathogenic avian influenza A(H7N2) virus in an animal shelter in 2016 suggests that exposure to cats infected with HPAI A(H5N1) virus might also pose a transmission risk to humans (*9*).

Although reported cases of infection of indoor cats with HPAI A(H5N1) viruses are rare, such cats might pose a risk for human infection. The source of HPAI A(H5N1) virus infection in these two cats is unknown; however, the cats’ owners worked on dairy farms and potentially had occupational exposures to HPAI A(H5N1)–positive dairy cattle or contaminated products or environments. Further research is necessary to evaluate the risk of fomite transmission and other types of transmission routes of HPAI A(H5N1) virus to cats. The two dairy workers described in this report did not use recommended PPE before their illnesses and could have been exposed to HPAI A(H5N1) virus. However, because neither dairy worker received testing for A(H5), whether cat 1A’s owner’s gastrointestinal symptoms or cat 2A’s owner’s ocular symptoms were because of HPAI A(H5N1) virus infection or a different etiology is unknown.

### Implications for Public Health Practice

Given the potential for fomite contamination, farmworkers are encouraged to consider removing clothing and footwear and to rinse off any animal byproduct residue (including milk and feces) before entering households.*** Veterinarians evaluating companion cats with signs of respiratory or neurologic illness in areas with HPAI A(H5N1) virus circulating in cattle or poultry or other animals are recommended to wear PPE when examining these animals or collecting specimens for influenza testing and to obtain occupational information from household members to help prevent unprotected exposures and guide coordinated One Health^†††^ (i.e., human, animal, and environmental) public health investigations of potential animal-to-human spread of HPAI A(H5N1) virus. Implementation of standard precautions for zoonotic disease prevention and CDC guidance for veterinarians at veterinary clinics can help limit the number of staff members exposed to sick animals potentially infected with pathogens, including HPAI A(H5N1) virus. Further, given the widespread outbreak in animals, including poultry and wild birds, throughout the United States, anyone who has occupational or recreational exposure should wear the recommended PPE when interacting with any potentially infected animals.^§§§^

## Footnotes

* Testing for avian influenza virus in cat specimens was performed using matrix PCR; subsequent testing for avian influenza virus H5 was done by PCR. No other testing was reported.

† All human specimens in this investigation (except one specimen from an MSU veterinary staff member who received testing only for influenza A) were tested with the Biofire FilmArray Torch System (https://www.biomerieux.com/us/en/our-offer/clinical-products/biofire-torch-system.html), which includes a panel of pathogens including adenovirus, coronaviruses (229E, HKU1, NL63, and OC43), SARS-CoV-2, human metapneumovirus, human rhinovirus/enterovirus, influenza A and B, parainfluenza (viruses 1–4), respiratory syncytial virus, *Bordetella pertussis*, *Mycoplasma pneumoniae*, *Chlamydia pneumoniae*, and *Bordetella parapertussis*.

§ 45 C.F.R. part 46, 21 C.F.R. part 56; 42 U.S.C. Sect. 241(d); 5 U.S.C. Sect. 552a; 44 U.S.C. Sect. 3501 et seq.

¶ Although the farm’s manager denied the presence of ill animals at the farm, the household member who worked on the dairy farm reported many of the barn cats on the premises had recently died.

** The owners in both households were not asked whether their pets ate raw cat food. This report investigates a detection of HPAI A(H5N1) in indoor domestic cats that were presumably exposed through their dairy farmworkers in May 2024, which preceded the recent detection of cats with HPAI A(H5N1) presumably exposed through commercially available contaminated raw pet food in late 2024.

†† Although oseltamivir postexposure prophylaxis of HPAI A(H5N1) is recommended for 5 days, longer duration can be considered if exposure is ongoing.

§§ The household contacts had a much more intense and longer exposure to the cats than did veterinary staff members and were provided antivirals as prophylaxis, not as treatment.

¶¶ Commercially available raw poultry cat food has been reported to test positive for HPAI A(H5N1) virus since this investigation concluded (http://publichealth.lacounty.gov/vet/docs/AHAN/AHAN_H5BirdFluDomesticCats_ConfirmedInRawPetFood_RawMilk_01132025.pdf). However, the genotype of clade 2.3.4.4b HPAI A(H5N1) virus identified in the cats in this investigation is different from the genotype that was identified in the commercially available cat food outbreak.

***
https://www.cdc.gov/bird-flu/prevention/farm-workers.html

†††
https://www.cdc.gov/one-health/about/index.html

§§§ https://www.cdc.gov/bird-flu/hcp/animals/index.html
